# In Pursuit of New Imprinting Syndromes by Epimutation Screening in Idiopathic Neurodevelopmental Disorder Patients

**DOI:** 10.1155/2015/341986

**Published:** 2015-05-27

**Authors:** Sonia Mayo, Sandra Monfort, Mónica Roselló, Silvestre Oltra, Carmen Orellana, Francisco Martínez

**Affiliations:** Unidad de Genética y Diagnóstico Prenatal, Hospital Universitario y Politécnico La Fe, Avenida de Campanar 21, 46009 Valencia, Spain

## Abstract

Alterations of epigenetic mechanisms, and more specifically imprinting modifications, could be responsible of neurodevelopmental disorders such as intellectual disability (ID) or autism together with other associated clinical features in many cases. Currently only eight imprinting syndromes are defined in spite of the fact that more than 200 genes are known or predicted to be imprinted. Recent publications point out that some epimutations which cause imprinting disorders may affect simultaneously different imprinted *loci*, suggesting that DNA-methylation may have been altered more globally. Therefore, we hypothesised that the detection of altered methylation patterns in known imprinting *loci* will indirectly allow identifying new syndromes due to epimutations among patients with unexplained ID. In a screening for imprinting alterations in 412 patients with syndromic ID/autism we found five patients with altered methylation in the four genes studied: *MEG3, H19, KCNQ1OT1*, and *SNRPN*. Remarkably, the cases with partial loss of methylation in *KCNQ1OT1* and *SNRPN* present clinical features different to those associated with the corresponding imprinting syndromes, suggesting a multilocus methylation defect in accordance with our initial hypothesis. Consequently, our results are a proof of concept that the identification of epimutations in known *loci* in patients with clinical features different from those associated with known syndromes will eventually lead to the definition of new imprinting disorders.

## 1. Introduction

Intellectual disability (ID) is a complex disease which affects 2% of our population. Known genetic and environmental causes are responsible for a large proportion of the cases; however the etiology in many patients remains unknown because of the elevated clinical and genetic heterogeneity. Deregulation of epigenetic mechanisms in brain development and neuronal plasticity may be associated with a wide spectrum of neurological and psychiatric disorders [1–4]. In fact, a growing number of syndromic forms of ID are caused by mutations in genes involved in epigenetic regulation as Sotos or Rett syndrome among others. However, these mutations only account for a small number of cases. There are evidences that aberrant epigenetic mechanisms play a role in autism and other neurodevelopmental disorders [5–7]. Also, genome-scale approaches to study the epigenetic alterations point out a possible association of global hypomethylation and different neurological disorders as schizophrenia or bipolar disorder [8, 9]. However, high-throughput methodology is expensive, time-consuming, and of complex and controversial interpretation in many occasions [10, 11].

Genomic imprinting is an epigenetic mechanism by which gene expression is regulated in a parent-of-origin-specific manner [12]. There are 95 proven and 114 predicted imprinted genes in the human genome (Geneimprint database). Furthermore, many of these genes are expressed in the central nervous system, among other tissues, and are predicted to act as transcriptional regulators in development. Nevertheless, the clinical consequences of the loss of function of these genes, due to mutation or epimutation, are largely unknown. Currently, there are 8 recognised imprinting syndromes associated with growth and behavioural disorders: Silver-Russell syndrome (SRS), Beckwith-Wiedemann syndrome (BWS), Prader-Willi syndrome (PWS), Angelman syndrome (AS), transient neonatal diabetes (TNDM), maternal uniparental disomy 14-like (UPD(14)mat) and UPD(14)pat-like syndromes, and pseudohypoparathyroidism 1B (PHP1B). The mechanisms that result in altered imprinted gene expression are diverse. Four different mechanisms have been described: large deletions or duplications of regions containing imprinted genes, DNA mutations in an imprinted gene, uniparental disomy, and epimutations. Each different cause is associated with varying recurrent risks; for example, epimutations or* de novo* deletions usually imply very low risk of recurrence for parents and other relatives, whereas some deletions and point mutations can have a 50% recurrence.

Recent publications claim that several genetic variants manifest a parent-of-origin effect in autism [6, 13, 14]. Moreover, it is increasingly evident that epimutations leading to imprinting disorders in some instances may affect not one but several imprinted* loci* throughout the genome, suggesting that imprinting-specific DNA-methylation may have been altered more globally due to unknown factors [15–21]. Phenotypic differences of these cases with the classical imprinting syndromes may be present or not and can be attributed to abnormal DNA-methylation elsewhere.

Based on these findings, we hypothesize that many of the imprinted genes of unknown clinical consequences may be responsible for neurodevelopmental disorders when epimutated, associated or not with other congenital anomalies. The detection of altered methylation patterns in known imprinted* loci* will allow the identification of new syndromes due to* multilocus* epimutations among patients with unexplained neurodevelopmental disorders. To asses this hypothesis, we searched for aberrant methylation at four imprinted* loci* (*SNRPN, H19, KCNQ1OT1, *and* MEG3*) in a series of 412 patients with intellectual disability using a methylation analysis affordable for any laboratory. We found five cases with alteration of methylation: two alterations in the methylation pattern of MEG3 as a consequence of paternal or maternal uniparental disomy for chromosome 14, one hypermethylation of H19 (due to paternal 11p duplication), one partial loss of methylation in KCNQ1OT1, and one partial loss of methylation in SNRPN.

## 2. Patients and Methods

### 2.1. Patient Samples

DNA samples of 412 patients were analyzed in this study. They were recruited for genetic investigation of unexplained ID and/or autism during more than 10 years (October 2001–July 2013). This research was carried out according to the principles of the Declaration of Helsinki. Informed consent, approved by the Hospital Ethics Committee, was obtained from all the parents of the children who participated in the study.

Genomic DNA was isolated from peripheral blood. Former samples (up to 2009) were extracted by the phenol extraction protocol described by [22]. Since 2010 the DNA extraction was performed using QIAamp DNA Mini Kit and the QIAcube automated extractor (QIAGEN, Hilden, Germany). DNA quality and concentration were measured using the NanoDrop ND-1000 Spectrophotometer (NanoDrop Technologies, Rockland, DE, USA) and were stored at −20°C.

The selection criteria of the patients, in addition to the intellectual disability or autism spectrum disorders (ASD), were the presence of congenital abnormalities, dysmorphic features, and/or a positive family history for neurodevelopmental disorders or congenital abnormalities. None of the patients had a specific genetic diagnosis when recruited. Genomic rearrangements' analyses by array CGH were performed in all these patients as part of our investigation. The methylation study was systematically carried out, as a blind test, that is, not taking into account previous genetic results. Once the analysis was performed, all the pieces of information were gathered together for the phenotype-genotype correlation.

### 2.2. Previous Tests

Genomic rearrangements were studied by oligonucleotide-based genome-wide array CGH (44K, G4426B; Agilent Technologies, Palo Alto, CA, USA), a targeted custom array for ID and autism (manuscript in preparation; Agilent Technologies), SNP-array (Affymetrix Genome-Wide Human SNP 6.0 Array, Santa Clara, CA, USA), MLPA (MRC-Holland), and/or FISH (telomeric commercial probes TelVysion, Vysis, Downers Grove, IL, USA), using the recommended protocols by the manufacturer with minor modifications [23–25].

The data related to array results discussed in this paper have been deposited in NCBI's Gene Expression Omnibus [26] and are accessible through GEO series accession number GSE62440 (http://www.ncbi.nlm.nih.gov/geo/query/acc.cgi?acc=GSE62440).

### 2.3. Methylation Test

Based on a multiplex amplification and quantification methylation test previously described in Martínez et al. [27], we performed a screening for DNA-methylation alterations in four differentially methylated regions (DMRs) associated with specific imprinting syndromes:* KCNQ1OT1* (11p15; BWS),* H19* (11p15; SRS and BWS),* SNRPN* (15q12; PWS and AS), and* MEG3* (14q32; UPD14pat and UPD14mat), in our series of patients.

50 ng of genomic DNA was digested with 10 units of the methylation sensitive enzyme HpaII (Fermentas), while another aliquot was used as undigested control. Both were incubated at 37°C for one hour, followed by heat inactivation at 94°C for 3 minutes. Undigested and digested DNAs were used as a template for a FAM-labelled multiplex PCR under semiquantitative conditions (see Supplementary Table 1 in Supplementary Material available online at http://dx.doi.org/10.1155/2015/341986). Resulting PCR products were analysed on an ABI-3130XL genetic analyser (Applied Biosystems) and each peak area was divided by the sum of all peak areas of that sample (relative area) and then normalized to the corresponding averaged relative areas obtained on control samples. Data analysis was performed in an Excel spreadsheet (Microsoft Office 2007).

### 2.4. Confirmation Tests

DNA samples with a relative value of methylation in the screening outside the 0.8–1.2 normal range were confirmed with alternative techniques.* KCNQ1OT1* and* H19* alterations were validated by methylation specific multiplex ligation-dependent probe amplification (MS-MLPA) using SALSA ME030; and* SNRPN* alterations were validated with SALSA ME028 (MRC-Holland, Amsterdam, Netherlands). The technical protocols and the analysis were performed as recommended by the manufacturer (MRC-Holland).

The possibility of a uniparental disomy was tested by segregation analysis of different microsatellite markers from the corresponding* loci* in the patients and their parents DNA samples (reagents and PCR conditions at Supplementary Table 2).

All genomic coordinates given below are based on Human Feb. 2009 assembly (GRCh37/hg19).

## 3. Results

In the screening for imprinting alterations in the 412 patients with neurodevelopmental disorders we have found five cases with different alterations of methylation.

### 3.1. Patient 1

**Figure 1:**
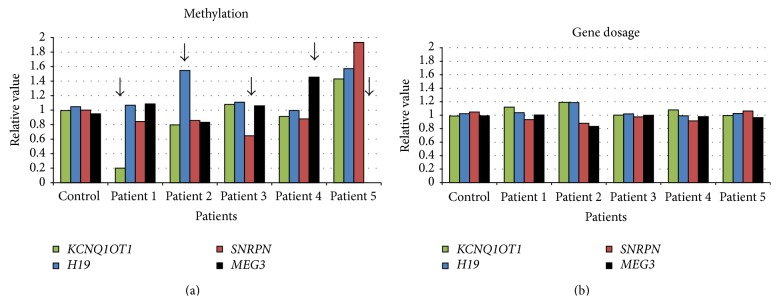
Methylation screening results. Representation of the relative value of methylation and gene dosage of the four imprinted regions (*KCNQ1OT1*,* H19*,* SNRPN*, and* MEG3*). A relative value within 1 ± 0.2 was considered in the normal range. The first case (left) represents a nonaltered patient. Subsequently, the results from the positive cases with different alterations of methylation are shown. A black arrow indicates the different alterations.

The patient presents an 80% loss of methylation at* KCNQ1OT1* with a normal gene dosage (Figure 1). The results were confirmed by MS-MLPA (SALSA ME030). Segregation analysis of chromosome 11 markers discarded the UPD as the genetic mechanism responsible of the altered methylation pattern. Screening for dosage alterations was performed by oligo-CGH-array with no relevant findings (GSM1527006).

This case was previously published with the clinical description of the patient [28]. In addition to motor and language delay and mild intellectual disability, he presents some clinical features resembling Sotos syndrome such as overgrowth, frontal bossing, sparse hair in the frontoparietal area, macrocephaly, and dolichocephaly.

### 3.2. Patient 2

This female patient shows hypermethylation of* H19* and an increased dosage of* H19* and* KCNQ1OT1* in chromosome 11 (Figure 1). Previous genetic analyses detected a complex rearrangement: a 3.1 Mb 11pter-p15.4 duplication and a 3.7 Mb 4pter-p16.2 deletion due to an unbalanced translocation inherited from her father (arr [hg19] 4p16.3(1-3,770,271) × 1 pat, 11p15.5p15.4(1-3,381,999) × 3 pat) (GSM1527007). Both results are in agreement (Table 1).

She was the first-born child of unrelated healthy parents, a 27-year-old mother and a 28-year-old father. She was born at term by normal delivery. Her birth weight was 3,360 g (75–50th percentile) and her length was 52 cm (90th percentile). Prenatal cytogenetic analysis was performed with normal results. On physical examination at 8 years of age, she presents hypotonia and some dysmorphic features as facial asymmetry, prominent forehead, hypertelorism, upslanting palpebral fissures, prominent nasal bridge, down-turned corners of the mouth, macroglossia, and dysmorphic ears. Congenital abnormalities include microcephaly, low-set hair, umbilical hernia, and tapering fingers. She also presents seizures, development delay (she walked at 4 years and did not speak at the age of the examination), and ID.

### 3.3. Patient 3

This case presents a 40% loss of methylation at* SNRPN* without alteration in the gene dosage (Figure 1) confirmed by MS-MLPA (SALSA ME028). Segregation analysis of chromosome 15 polymorphic markers discarded the paternal UPD. Besides, analysis by a custom array CGH, targeted to more than 400 candidate genes and a genomic backbone of 370 Kb resolution, did not yield any pathogenic copy number variant (GSM1527009).

The patient is the second child of nonconsanguineous healthy parents of 34 and 33 years. She was born in the 39th week of gestation with a birth weight of 3,255 g (50–75th percentile) and a length of 52 cm (90th percentile). At the age of seven years and 10 months she weighed 38 kg (>97th percentile) and her height was 135 cm (>97th percentile). Clinical examination noted facial dysmorphisms: hypertelorism, strabismus, dysmorphic nose, short philtrum, micrognathia, and low-set and posteriorly rotated ears. She has global developmental delay (sitting at 14 months and walking at 24 months) and ID. She only spoke single words since the age of 3. Brain magnetic resonance imaging (MRI) and electroencephalography (EEG) were normal.

### 3.4. Patient 4

The male patient showed a hypermethylation at* MEG3* without alteration in the gene dosage (Figure 1). Segregation analysis of polymorphic markers at chromosome 14 indicated a paternal uniparental disomy (UPD(14)pat) as the pathogenic mechanism of this alteration. Further studies also showed a duplication in chromosome 4 (4p16.3) of 146 Kb inherited from his father (arr [hg19] 4p16.3(1,694,662-1,841,014) × 3 pat) (GSM1527008) (Table 1).

He was the first-born child of unrelated healthy parents, a 22-year-old mother and a 27-year-old father. He was born in the 36th week by caesarean section. During the pregnancy he presents polyhydramnios, short femur, and omphalocele. His birth weight was 3,675 g (>90th percentile), his length was 49 cm (75–90th percentile), and his neonatal OFC was 36 cm (>90th percentile). He had neonatal hypotonia and feeding difficulties with an Apgar score of 2/5. On physical examination at 10 years, his height and weight were 132 cm (25th percentile) and 29 kg (25th percentile), respectively, and the hypotonia remained. Facial dysmorphism is seen in the form of prominent forehead, divergent strabismus, ptosis and upslanting palpebral fissures, prominent nasal bridge, thick lips, absence of some teeth, prognathism, and dysmorphic ears. Congenital abnormalities include, in addition to the omphalocele, surgically corrected at birth, tracheomalacia, patent ductus arteriosus, scoliosis, inguinal hernia, brachydactyly of the third, fourth, and fifth metacarpals, and valgus and flat feet. He presents a psychomotor development delay (walked and said his first words at 3 years and spoke simple sentences at 4) and is moderately mentally disabled. He also has nightmares and aggressiveness towards others and himself. Brain MRI and EEG results were normal.

### 3.5. Patient 5

This case presents a complete loss of methylation at* MEG3* (14q32.2) without alteration in the gene dosage (Figure 1). By segregation analysis of polymorphic markers at chromosome 14, a maternal uniparental disomy (UPD(14)mat) was evidenced. Besides, previous assays indicated a* de novo* duplication at chromosome 14 of 5.7 Mb previously published [29] (arr [hg19] 14q11.2(19,002,011-24,748,363) × 3 dn) (GSM1527005) (Table 1). Familial segregation analysis of markers inside the duplicated area indicated the presence of two copies of the maternal alleles and one copy of the paternal allele, and FISH analysis confirmed an* in situ* duplication. Therefore, the patient inherited the two 14 homologues from her mother. Additionally, the duplication corresponds to the insertion of the subcentromeric region from one paternal chromosome into one maternal chromosome.

His main clinical features, previously described by Monfort et al. [29], besides psychomotor delay and mild ID, are short stature (<3rd centile) of prenatal onset, hypogenitalism, and some dysmorphic signs such as iris coloboma at the left eye, a bulbous nose, short philtrum, thin lips, clinodactyly and bilateral partial syndactyly between toes II and III, and micrognathia.

## 4. Discussion

With this study we have been able to achieve or complete the diagnosis in five patients with ID: two alterations in the methylation pattern of* MEG3* as a consequence of paternal or maternal uniparental disomy for chromosome 14, one hypermethylation of* H19* (due to a paternal 11p duplication), one partial loss of methylation in* KCNQ1OT1*, and one partial loss of methylation in* SNRPN*.

In addition to motor and speech delay and ID, growth anomalies (birth weight, birth length, and/or height at examination ≤10 centile or ≥90 centile) were present in all the five cases with different methylation anomalies detected by this screening (Table 1). By comparison, 46% of the patients in the whole series share all these symptoms. Other recurrent features in these five patients were macro- or microcephaly (2 cases), hypotonia (2 cases), hypertelorism (3 cases), and strabismus (2 cases).

Although patient 2 presents a genomic rearrangement responsible of the imprinting alteration, the result of the methylation test validates our strategy to detect imprinting alterations in specific* loci*. It is worth noting that the phenotype in the patient would be the result of two concomitant syndromes, Beckwith-Wiedemann syndrome, due to the 11p15 duplication, and the Wolf-Hirschhorn syndrome due to the 4p16 deletion, similarly to other patients reported elsewhere [30–32]. It has been suggested that the number of cases with a CNV at the critical region of BWS could be higher than suspected and that the methylation analysis in those cases can be insufficient to provide accurate clinical diagnosis and genetic counselling.

Patients 4 and 5 showed an alteration in the methylation of* MEG3* due to a UPD(14) in both cases, compatible with the clinical features of the patients [33–36]. Accordingly, these results lead to a reevaluation of the 5 Mb duplication at 14q11.2, previously considered as the cause of the clinical phenotype in patient 5 [29]. The point is that all the clinical features can now be ascribed to the maternal UPD(14), while the duplication should be considered a variant of unknown significance. Also it is worth noting that patient 4 lacks thorax deformities, which are a hallmark of upd(14)pat.

Finally the most interesting results of this study are the diagnosis of two patients with idiopathic ID. Patient 1 shows a partial loss of methylation in* KCNQ1OT1* in spite of the absence of the typical features of Beckwith-Wiedemann syndrome, such as abdominal wall defects, macroglossia, hemihypertrophy, and coarse facial features; conversely he presents a Sotos-like syndrome, with the characteristic facial gestalt (downslanting palpebral fissures and pointed chin), neonatal hypotonia, large hands, or cardiac anomalies. This association was previously described by Baujat's group in a series of Sotos-like patients with no alteration in* NSD1* [37]. On the other hand, patient 3 shows a partial loss of methylation in* SNRPN* without any other known genetic alteration. Although some clinical features of this patient might be compatible with a mild Angelman syndrome (intellectual disability and motor and speech delay), the lack of the cardinal characteristics of this disease, such as microcephaly, ataxic movements, seizures, or a distinctive behaviour [38], and more significantly the presence of other features not associated with Angelman syndrome, such as the overgrowth and some facial dysmorphic features (hypertelorism, short philtrum, micrognathia, and low-set ears), allow us to classify this case as another syndrome different to AS.

Both cases present a partial loss of maternal methylation. Several groups have demonstrated* multilocus* methylation defects in specific imprinting syndromes as in BWS [16, 17] or in TNDM [39]. Based on this, Azzi et al. [18, 40] proposed a* multilocus* loss of methylation condition where the dominant phenotype in those cases might be determined by the* locus* more demethylated. In our cases, the partial demethylation does not explain the phenotype observed in patients 1 and 3 so as Girardot et al. [41] suggested, other unknown imprinted* loci* might be affected.

Given that the pattern of differential methylation may be tissue-specific and/or time-specific, a high-throughput analysis of all the differentially methylated regions (DMRs) would not be necessarily useful in genomic DNA from blood cells. The unavailability of some tissue material as brain hampers the finding of new imprinting disorders that might be associated with ID as in those cases.

Also, one possible explanation for a global affectation at imprinting* loci* could be the presence of mutation at genes coding for transacting factors involved in imprinting establishment. In this sense, mutations in some genes lead to* multilocus* loss of methylation:* ZFP57* in TNDM patients [15],* NALP7* and* C6orf221* in familial biparental hydatidiform mole [42, 43], or* NLRP2* in a family with BWS [44]. However, a prerequisite to perform whole genome or exome sequence analysis would be the recruitment of clinically and epigenetically homogenous series of patients in order to interpret the results.

In a similar study to this work, although focused on patients with putative or confirmed imprinting disorders, 22% of patients with molecular diagnosis of an imprinting syndrome showed methylation anomalies in other* loci*, with no overt clinical consequences in some cases [21]. Other patients (more specifically BWS and SRS patients) associated developmental delay and other unusual congenital anomalies. In our complementary approach, focused on syndromic intellectual disability and ASD, we also found patients with methylation anomalies not associated with the corresponding syndrome but most probably reflecting new imprinting disorders with* multilocus* methylation anomalies. The clinical and epigenetic features that have in common the patient 1 and those reported by Baujat and colleagues [37] fully agree with this hypothesis.

## 5. Conclusion

In summary, our findings show that the complex etiology of neurodevelopmental disorders not only is limited to genetic factors, but also may be epigenetic changes that constrain or modify the phenotype of the patients. In conclusion, we found evidences of new* multilocus* imprinting syndromes in two patients, although further studies, not easily affordable nowadays, would be necessary to confirm this hypothesis.

## Supplementary Material

Table 1: PCR conditions, primer sequences and reagents employed for the methylation test. Note that the PCR are mounted in duplicate with and without the addition of HpaII methylation-sensitive enzyme.
Table 2: PCR conditions, primer sequences and reagents employed for amplification of microsatellite markers used to evaluate the uniparental disomy by segregation analysis in the family (patient and parents).

## Figures and Tables

**Table 1:** Genetic and epigenetic alterations in the patients and correlation with their phenotype and the epigenetic syndrome associated.

Gene	*KCNQ1OT1 *	*H19 *	*SNRPN *	*MEG3 *	

OMIM#	^*∗*^604115	^*∗*^103280	^*∗*^182279	^*∗*^605636	
Cytoband	11p15		15q12	14q32	
Methylated allele	Maternal	Paternal	Maternal	Paternal	
Disease	BWS		AS	UPD14(pat)	UPD14(mat)
OMIN#	#130650		#105830	#608149	
Epigenetic alterations	Hypomethylation	Hypermethylation	Hypomethylation	Hypermethylation	Hypomethylation

Patient	1	2	3	4	5

Genetic alterations	—	arr 4p16.3(1-3,770,271) × 1 pat, 11p15.5p15.4(1-3,381,999) × 3 pat	—	UPD(14)pat arr 4p16.3(1,694,662-1,841,014) × 3 pat	UPD(14)mat arr 14q11.2(19,002,011-24,748,363) × 3 dn

Clinical features^1^	*Prenatal and postnatal overgrowth* Macrocephaly Dolichocephaly Frontal bossing High hairline Motor delay Speech delay Intellectual disability Behavioural problems	*Prenatal overgrowth* *Microcephaly* Facial dysmorphism and *asymmetry* *Macroglossia * *Umbilical hernia* *Hypotonia * Motor delay Speech delay Intellectual disability Seizures	Prenatal and postnatal overgrowth Facial dysmorphism *Motor delay* *Speech delay* *Intellectual disability *	Prenatal and postnatal overgrowth *Hydramnios* *Omphalocele* Feeding difficulties Facial dysmorphism *Inguinal hernia * Scoliosis Brachydactyly Hypotonia Motor delay Speech delay Intellectual disability Behavioural problems	*Prenatal and postnatal growth retardation* Dysmorphic features Hypogenitalism Stagnation of pubertal development *Psychomotor delay * *Speech delay* Intellectual disability

Diagnosis	Sotos-like syndrome	Wolf-Hirschhorn syndrome Beckwith-Wiedemann syndrome		UPD(14)pat	UPD(14)mat

^1^Italic features are present in the characteristic phenotype associated with the methylation alteration present in each patient. Patient 1 has been previously published at Mayo et al. [28], and clinical features of patient 5 are indicated in Monfort et al. [29].

BWS, Beckwith-Wiedemann syndrome; SRS, Silver-Russell syndrome; PWS, Prader-Willi syndrome; AS, Angelman syndrome; UPD(14)pat, paternal uniparental disomy for chromosome 14; UPD(14)mat, maternal uniparental disomy for chromosome 14.
