# Supplementary material for: In Pursuit of New Imprinting Syndromes by Epimutation Screening in Idiopathic Neurodevelopmental Disorder Patients
Source: Biomed Res Int. 2015 May 27;2015:341986. doi: 10.1155/2015/341986 (PMC4461700; doi:10.1155/2015/341986)
Supplement: Supplementary file 1 — Table 1: PCR conditions, primer sequences and reagents employed for the methylation test. Note that the PCR are mounted in duplicate with and without the addition of HpaII methylation-sensitive enzyme. Table 2: PCR conditions, primer sequences and reagents employed for amplification of microsatellite markers used to evaluate the uniparental disomy by segregation analysis in the family (patient and parents). [file 341986.f1.pdf]

Supplementary table 1: Material and method used in the methylation test.

	Reagents	Amount		Primer sequence	Size
	H_2O	9 µl			
	Master Mix 2X	12 µl (1X)			
	DMSO	1.25 µl (5%)			
**Primers**	SNRPN_F* (50uM)	312 nM		AGGTCATTCCGGTGAGGGAGG	490 bp
**Primers**	SNRPN_R (50uM)	312 nM		ACCGCAGACACCCGCAATAGG	
**Primers**	H19_F* (50uM)	250 nM		GGCAACATGCGGTCTTCAGAC	420 bp
**Primers**	H19_R (50uM)	250 nM		TCCGGAGACAGGGCTGAGCA	
**Primers**	MEG3_F* (50uM)	250 nM		TCCTGACATGTTGCAGTCTTG	386 bp
**Primers**	MEG3_R (50uM)	250 nM		CTCCACAACACCCGAAGCCAC	
**Primers**	KCNQ1OT1_F* (50uM)	187 nM		CCTGTCATTGGCCGAAAGAGTC	366 bp
**Primers**	KCNQ1OT1_R (50uM)	187 nM		GTTCGAGGGTCGCTGCAGC	
	HpaII[1] /H2O	10 U	1 µl		
	DNA	50 ng			
	TOTAL	25 µl			
		Digested	Undigested		

PCR program
37ºC X 60 min
94ºC X 1 min
96ºC X 40 s (X 28 cycles)
59ºC X 1 min (X 28 cycles)
72ºC X 1 min (X 28 cycles)
72ºC X 7 min

* labelled with FAM

[1] New Onglad Biolabs

F: forward; R: reverse

Supplementary Table 2: Segregation microsatellite markers used to evaluate a uniparental disomy as the cause of the methylation alteration. PCR reagents and programs, as well as sequence and size of each amplicon used in the PCR reactions. All reagents are from Roche except from Taq polymerase that is from

Marker (location)	Sequence	PCR reagents	PCR program	Size (bp)	Chr
D11S1984 (1.57Mb)	GGGTGACAGAGCAAAATTCT<br>ACACCTGGATCTTGGACTCA	1µl gDNA (100ng/µl)<br>1X PCR reaction Buffer<br>2.5U Taq polimerase<br>480µM dNTPs<br>1µM Primer F<br>1µM Primer R<br>25µl V_f	3min 94ºC<br>30s 94ºC<br>30s 60ºC-0.5ºC/cy<br>30s 72ºC<br>(10 cycles)<br>30s 94ºC<br>30s 55ºC<br>30s 72ºC<br>(20 cycles)<br>7min 72ºC	170-202	11
D11S2362 (4.91Mb)	TGGACTATAGGACCCCCTTC<br>GAGAACAGCCTGTCACACCT			209-230	
D11S1999 (10.72Mb)	TACATGGCAGCAGGCATATA<br>GAGTAAACAAGATTGCTAGATAGGC			109-137	
D14S288 (44.10Mb)	AGCTAGACTCTGCCATAAACA<br>TGGAGACAGGAACAACACAC			189-209	14
D14S68 (88.63Mb)	GAGAGGTGGTTTTCAGTGGT<br>TCAGGGATAGTTGGTGGGTA			148-172	
D14S1006 (101.18Mb)	TTCCACAGGGCAAGCAGTA<br>TTCTGGCAAAACCCAACC			121-155	
D14S985 (101.30Mb)	CAGTGTGACCTTAAACAAGTCG<br>CCTGTGGGGTAGATACACGA			118-142	
D14S72[1] (21.37Mb)	TGTAAAGTTTTGTACATGGTGTAAT<br>TCCTAACATTCTGCTACCCA			257-271	
D14S1023[1] (21.44Mb)	TGCATTTCCCGTAGACATT<br>GACTCTTGTAGTTCTTTGAAGCC			93-109	
D14S990[1] (23.59Mb)	GTCCACTTGGTCATGGAAAC<br>AAGTTGCACTGTGACTGGG			135-161	
D15S1035 (22.91Mb)	CACCCCCATGCAGAGTGAG<br>CCAAAGGCCAAGACCTGCC			175-265	15
D15S11 (24.09Mb)	GACATGAACAGAGGTAAATTGGTGG<br>GCTCTCTAAGATCACTGGATAGG			243	
D15S122 (25.68Mb)	GATAATCATGCCCCCCA<br>CCCAGTATCTGGCACGTA			143-159	
GABRB3 (26.74Mb)	CTCTTGTTCCTGTTGCTTTCAATACAC<br>CACTGTGCTAGTAGATTCAGCTC			180-200	

[1] Markers used to confirm duplication of patient 5 and the paternal origin of the alteration.

F: forward; R: reverse; V_f: final volumen
